# Prognostic Role of MicroRNA-181a/b in Hematological Malignancies: A Meta-Analysis

**DOI:** 10.1371/journal.pone.0059532

**Published:** 2013-03-22

**Authors:** Shenglong Lin, Lili Pan, Shicheng Guo, Junjie Wu, Li Jin, Jiu-Cun Wang, Shaoyuan Wang

**Affiliations:** 1 Department of Hematology, Fujian Medical University Union Hospital, Fuzhou, Fujian Province, China; 2 National Ministry of Education Key Laboratory of Contemporary Anthropology and State Key Laboratory of Genetic Engineering, School of Life Sciences, Fudan University, Shanghai, China; 3 Department of Pneumology, Changhai Hospital of Shanghai, Second Military Medical University, Shanghai, China; University of Barcelona, Spain

## Abstract

**Background:**

Emerging evidence has shown that miRNAs participate in human carcinogenesis as tumor suppressors or oncogenes, and have prognostic value for patients with cancers. In recent years, the miR-181 family was found dysregulated in a variety of human cancers and significantly associated with clinical outcome of cancerous patients. MiR-181a and miR-181b (miR-181a/b) were the most investigated members in the family. However, the results of miR-181a/b from different studies were inconsistent. Therefore, we performed a meta-analysis to summarize all the results from available studies, aiming to delineate the prognostic role of miR-181a/b in human cancers.

**Methods:**

The identified articles were retrieved from the two main on-line databases, PubMed and EMBASE. We extracted and estimated the hazard ratios (HRs) for overall survival (OS), which compared the high and low expression levels of miR-181a/b in patients of the available studies. Each individual HR was used to calculate the pooled HR.

**Results:**

Eleven studies of 1252 patients were selected into the final meta-analysis after a strict filtering and qualifying process. Fixed model or random model method was chosen depending on the heterogeneity between the studies. The subgroup analysis showed that high expressed miR-181a/b could prolong OS in patients with hematological malignancies rather than low expression level (HR = 0.717, P<0.0001). But the expression of miR-181a/b was not significantly relative to OS in patients with various cancers (HR = 0.861, p = 0.356).

**Conclusion:**

Our study indicates that the expression level of miR-181a/b is significantly associated with OS in hematological malignancies and can be an important clinical prognostic factor for those patients.

## Introduction

MicroRNAs (miRNAs) represent a class of highly conserved and small (average of 22 nucleotides) noncoding RNAs which can regulate gene expression and sequentially modulate various biological processes. MiRNAs were first discovered by the laboratory of Victor Ambrose in 1993 [1], and the knowledge of their critical roles in regulating proliferation, differentiation, apoptosis, development, metabolism and immunity has been greatly advanced recently. Circumstantial evidence has indicated the potential involvement of several miRNAs in tumorigenesis, after the first report that miR-15 and miR-16 were frequently deleted and/or downregulated in B-cell chronic lymphocytic leukemia in 2002 [2], [3]. A meta-analysis performed by Fu et al. [4] showed that elevated miR-21 expression was significantly associated with poor survival in patients with various types of carcinomas. Hence, miRNAs might act as oncogenes or tumor suppressors and they could play a potential role as diagnostic and prognostic biomarkers of cancers.

Like protein coding genes, miRNA sequences can be grouped into families and the relationship between their structures and functions can be learnt from multiple sequence alignments in miRNA families. However, the base-paired secondary structure is often conserved in miRNAs, rather than the conservation or similarity in primary sequences as in proteins [5]. A miRNA family usually has several members which are different in 1–2 nucleotides only. MiR-181 family is one of those miRNA families, which generally express in 70 species and various human cancers [6]. This family includes 4 members (miR-181a, miR-181b, miR-181c and miR-181d) and they are highly conserved in the seed-region sequence and RNA secondary structure.

Among them, miR-181a and miR-181b (miR-181a/b) which locate on the same loci of chr1q31.3 and chr9q3.33 are the most studied. Ciafre et al. [7], firstly reported that expression of miR-181a/b was significantly downregulated in primary glioblastomas and human glioblastoma cell lines compared to normal brain tissue, by using microarray and northern blot analysis. Thereafter, miR-181a/b was discovered abnormally expressed in various cancers including solid tumors and hematological malignancies. As in glioblastoma, significant down-regulation of miR-181a level was also observed in squamous lung cell carcinoma (SQCC), oral squamous cell carcinoma (OSCC) and non-small-cell lung cancer (NSCLC) [8]–[10]. However, miR-181a was significantly overexpressed in MCF-7 breast cancer cells and hepatocellular carcinoma (HCC) cells [11]. Other studies also reported that miR-181a had different expression levels in hematological malignancies. It is upregulated in acute myeloid leukemia (AML), especially in M1 and M2 subtypes, and myelodysplastic syndromes (MDS) [12], [13], but downregulated in multiple myeloma (MM) and chronic lymphocyte leukemia (CLL) [14], [15]. Notably, miR-181b has the same expression pattern as miR-181a in human cancers. Consider that the seed region of miR-181a/b is highly aligned and most of their predicted targeted genes are overlapped (Figure S1), miR-181a/b might co-express and play critical roles together in human cancers. Since the results of the present studies are inconsistent, it is unclear that miR-181a/b acts as oncogene or tumor suppressor. However, we can find some clues from several clinical studies investigating miR-181a/b as a prognosis factor in patients with cancers. Therefore, this literature review and meta-analysis were carried out to summarize the studies globally.

## Methods

### Guidelines and Search Strategy

This meta-analysis was performed by the guidelines of PRISMA (Preferred Reporting Items for Systematic Reviews and Meta-Analysis) Statement issued in 2009 (Checklist S1). We carefully searched online database PubMed and EMBASE to identify relevant published studies from Jan 1st, 1993 to Oct 5, 2012. For PubMed, the contextual query language (CQL) was “*(mir-181[Title/Abstract]) OR (microRNA-181[Title/Abstract]) OR (mir-181a[Title/Abstract]) OR mir-181b[Title/Abstract]”*; for EMBASE, the CQL was *“(mir-181 or microRNA-181 or mir-181a or mir-181b).ti,ab”*. The references manager software EndNote(X5, Bld5478) was used to check out duplications. The candidate studies should follow these inclusive criteria: (i) it studied miR-181a/b in any type of human cancers; (ii) it measured miR-181a/b expression in human samples; (iii) it investigated the association between miR-181a/b and survival outcome. Further, the candidate articles were manually screened by 2 authors (S Lin and L Pan) independently and were excluded if they were: (i) review articles or letters; (ii) non-English articles; (iii) investigation of a set of miRNAs but not miR-181a/b alone; (iv) nondichotomous miR-181a/b expression levels; (v) absent of key information such as hazard ratio (HR), 95% CI and *P* value. We also e-mailed the authors of some studies for addition information and data needed for our meta-analysis. The entire process was supervised by the third part (S Wang). Any disagreements were resolved immediately by four authors (S Lin, L Pan, S Guo and J Wu) after discussion.

### Quality Assessment and Data Extraction

**Figure 1:**
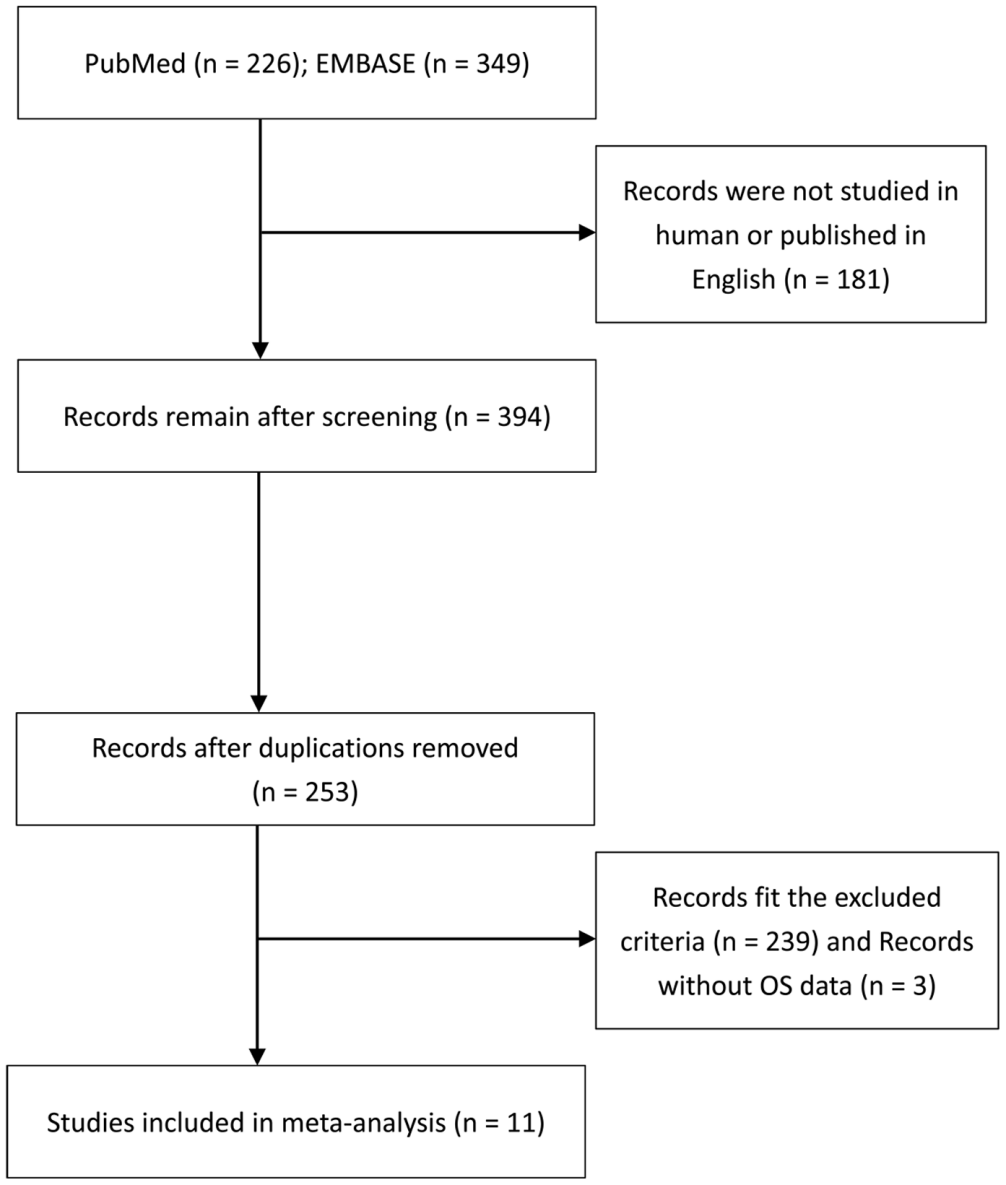
Flow diagram of the studies identification and selection.

The quality of all eligible studies was systematically assessed. The key components of a qualified study should include the followings: (i) clear definition of the study population; (ii) clear definition of the type of carcinoma; (iii) clear definition of the study design; (iv) clear definition of the outcome assessment; (v) clear definition of the measurement method of miR-181a/b; (vi) clear definition of the cut-off of miR-181a/b expression and (vii) sufficient period of follow-up time. The study lacks any point mentioned above will be excluded aiming to increase the reliability of the meta-analysis. A flowchart of the studies identifying process is presented in Figure 1. The following information was carefully deprived from the full texts of eligible articles: (i) publication details: first authors’ surname, publication year; (ii) characteristics of studies: origin country, sample size and tumor types; (iii) miR-181a/b assessment methods and the cut-off definition; and (iv) HR of miR-181a/b expression for overall survival (OS) as well as corresponding 95% confidential interval (CI) and P value. If the HR and CI were not reported directly, the total observed death events and the numbers of patients in each group were extracted to calculate HR and its variance indirectly [16]. If only Kaplan-Meier curves are available, data was extracted from the graphical survival plots. In this case, after dividing the time axis into non-overlapping intervals, log HR and its variance for each interval were calculated. These estimated values were combined in a stratified manner to obtain the overall HR and 95% CI [16]. We presumed that miR-181a and miR-181b may have the same effect on patients’ survival. In the studies which reported the HR data of miR-181a and miR-181b respectively in a same set of patients, the combined HR was estimated by simply taking the square-root of multiplying two HR data. If the author reported both univariate analysis and multivariate analysis to get the HR, the result of multivariate analysis including other variables should be preferably taken because it could be more accurate.

### Statistical Analysis

Firstly, HR with 95% CI was used to combine the pooled data. The statistical heterogeneity of studies was tested with the chi-square based Q-test, and absence of heterogeneity across studies was identified, then the fixed-effects model (the Mantel-Haenszel method) was used. Otherwise, the random effects model (the DerSimonian and Laird method) was performed. We also quantified the effect of heterogeneity using I^2^ statistic measuring the degree of heterogeneity. I^2^ value ranges from 0% to 100% (I^2^ = 0–25%, no heterogeneity; I^2^ = 25–50%, moderate heterogeneity; I^2^ = 50–75%, large heterogeneity; I^2^ = 75–100%, extreme heterogeneity) [17]. Secondly, evidence of publication bias was analyzed by the methods of Begg plots and Egger test (p<0.05 was considered representative of statistically significant publication bias). Finally, sensitivity analysis was carried out by investigating the influence of a single study on the overall HR. All of the analyses were carried out using STATA v11.0 (Stata Corp., College Station, TX).

## Results

Data were extracted from 11 studies with a total of 1252 patients from United States, China, Japan and Chinese Taiwan [10], [18]–[27]. All of them were retrospective in design. The types of cancers in these studies included solid tumors (colon cancer, NSCLC, OSCC, astrocytoma, gastric cancer and breast cancer) and hematological malignancies (cytogenetically normal AML, cytogenetically abnormal AML and CLL). Most of the studies used quantification real-time PCR to measure the expression level of miR-181 (TaqMan: 6 and Stem-loop: 2), and others used microarray method. Two studies both investigated 2 independent populations as a training set and a validation set [19], [24]. Li et al. and Zhu et al. examined MiR-181a and miR-181b respectively in the same population [19], [27], whereas Yang et al. studied the patients with both miR-181a and miR-181b overexpression [26]. Notably, the cut-off of miR-181a/b were different in the studies, applying median value in 6 studies, and the mean, the highest tertile, the highest value of 95% confidence interval as well as 3-fold in other studies. (Table S1).

**Figure 2:**
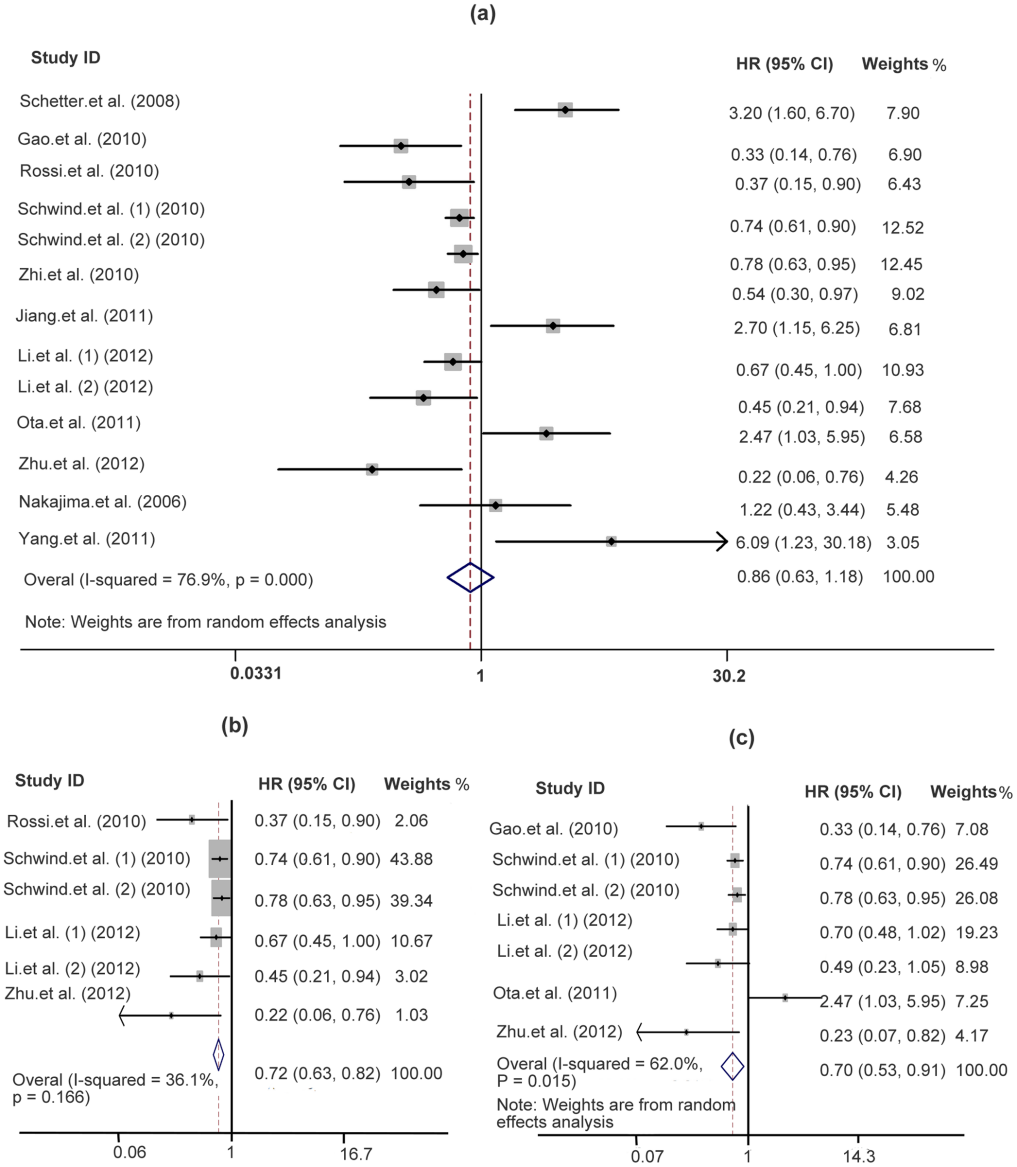
Forest plots of studies evaluating HR of overall survivals comparing high and low miR-181 expression. (a) Analysis of miR-181a/b expression in a variety of cancers, (b) analysis of miR-181a/b expression in hematological malignancies, (c) analysis of miR-181a expression in a variety of cancers.

**Figure 3:**
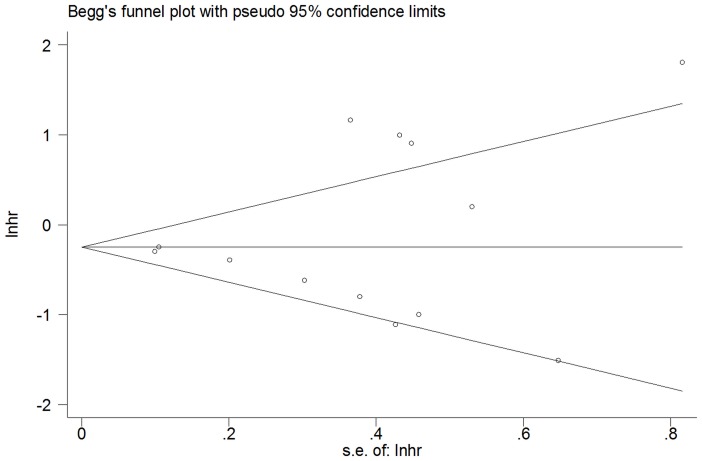
Begg’s funnel plot for publication bias analysis. Each point represents a separate study, lnhr is natural logarithm of HR, and horizontal line represents the mean effect size.

**Figure 4:**
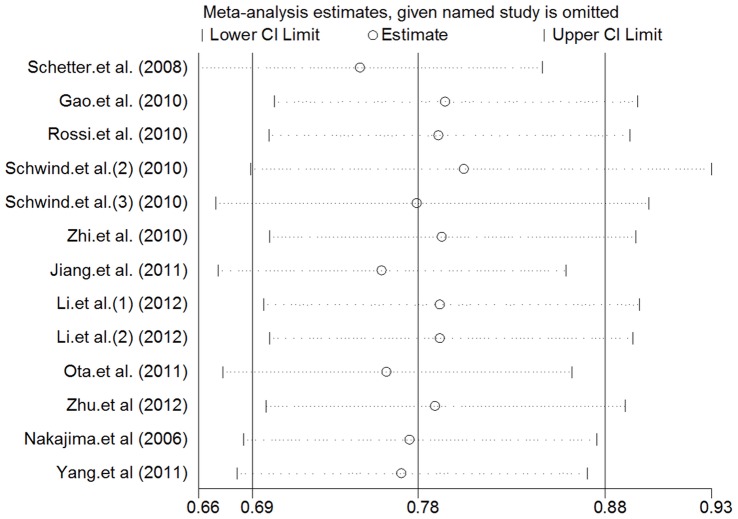
Sensitivity analysis. The middle vertical axis represents the pooled HR and the 2 vertical axes indicate the corresponding 95% CI. Each hollow circle represents the pooled HR when the left study was omitted in this meta-analysis, and the 2 ends of every broken line indicate the 95% CI.

Table 1 shows the main results of this meta-analysis. At first, we performed analysis of miR-181a/b expression and OS in a variety of cancers and it appeared extreme heterogeneity (I^2^ = 76.9%, p<0.0001) between the studies, so that a random effects model was applied to calculate a pooled HR (0.86, 95% CI: 0.629–1.184, p = 0.356) which was not statistically significant. And next, the subgroup analysis of hematological malignancies (n = 566) was carried out. The results showed only moderate heterogeneity between the studies of hematological malignancies (I^2^ = 36.1%, p = 0.166) and the pooled HR was more significant than any single HR of each study (0.717, 95% CI: 0.631–0.816; p<0.0001). Another subgroup analysis of miR-181a (n = 818) showed that the large heterogeneity existed (I^2^ = 62%, p = 0.015) and the pooled HR was statistically significant (0.698, 95% CI: 0.532–0.914; p = 0.009). Both pooled HRs <1 indicated that downregulated miR-181a and miR-181a/b may be associated with poor overall survival outcome in various cancers and hematological malignancies respectively (Figure 2). Finally, publication bias of the included studies was evaluated by Begg plots and Egger test. As shown in Figure 3, the Begg plots were almost symmetric and the Egger’s regression intercept was 0.509. There was no evidence for significant publication bias in this meta-analysis. Meanwhile, the sensitive analysis was performed by omitting one study at each time to measure its effect on the pooled HR. As presented in Figure 4, no individual study influenced the overall HR dominantly.

**Table 1:** Main results of meta-analysis.

		Heterogeneity analysis				
	Patients	Q test	P	Pooled HR	95% CI	P
**miR-181a/b** a	1252	76.90%	<0.0001	0.861	0.629–1.184	0.356
**miR-181a/b** b	566	36.10%	0.166	0.717	0.631–0.816	<0.0001^d^
**miR-181a** c	818	62.00%	0.015	0.698	0.532–0.914	0.009

a Analysis of the association of miR-181a/b and OS in a variety of cancers;

b Subgroup analysis of the association of miR-181a/b and OS in hematological malignancies;

c Subgroup analysis of the association of miR-181a and OS in a variety of cancers;

d The P value was calculated using the fixed-effects model (the Mantel-Haenszel method).

## Discussion

The present meta-analysis indicated that downregulated miR-181a/b could predict poor OS in patients with hematological malignancies, although the expression level of miR-181a/b was not significantly relative to OS in patients with various cancers. However, it should be circumspect to make a verdict of the association with miR-181a/b and human cancers, because there are still several issues should be considered. First, since the number of studies for each type of human cancers was less than 5, it might weaken the reliability of our results. A well-designed clinical study with large cases of each specific cancer should be performed in the future to validate the relationship between miR-181a/b expression level and prognosis of cancerous patients. Second, dislike oncogenes or tumor suppressor genes, miRNAs are generally associated with tumorigenesis through regulating the expression of hundreds of targeted mRNAs. Whether miR-181a/b is oncogene or tumor suppressor depends on which targeted genes are dominantly under the family’s control. Third, the precondition of our study is that miR-181a and miR-181b are co-expressed in cancers and playing an important role together in tumorigenesis. However, the subgroup analysis showed that low expression level of miR-181a, but no miR-181b (data no show), was significantly relative to poor survival outcome in patients. The similarity in primary sequences between miRNAs is not equal to the similarity in their functions. For instance, miR-181a and miR-181c have only one-nucleotide difference in their mature miRNA sequences, but only miR-181a can promote CD4 and CD8 double-positive (DP) T cell development, when ectopically expressed in thymic progenitor cells. The distinct activities of miR-181a and miR-181c are largely determined by their unique pre-miRNA loop nucleotides [28]. Although the seed region of miR-181a and miR-181b is highly aligned and most of their predicted targeted genes are overlapped, they might act differently in different kinds of cancers. Future study of combination and separation of miR-181a/b should be performed.

We also concede that there are several limitations in our meta-analysis. First, the heterogeneity existed in our meta-analysis and was probably due to the differences in baseline demographic characters of population, the tumor types, the disease stages, the cut-off value of miR-181 expression, the duration of follow-up, etc. When we divided the studies into solid tumors and hematological malignancies, the heterogeneity was markedly reduced. Second, although there was no significant evidence of publication bias in this meta-analysis, cautions should be taken because only studies published in English were selected, which could definitely cause language bias. And the tendency for journals to publish positive results could also make certain bias.

In recent years, miR-181 family has been found associated with tumorigenesis. In differentiated mouse embryonic stem cells (ESCs), miR-181a is one of the miRNAs that post-transcriptionally downregulate and maintain the low protein expression of silent mating-type information regulation 2 homologue 1 (SIRT1), which regulates processes such as transcription, apoptosis and muscle differentiation by deacetylating key proteins [29]. Studies also reported that miR-181a is frequently down-regulated in OSCC and may function as an OSCC suppressor by targeting on K-ras [9]. Likewise, miR-181b can enhance matrix metallopeptidases (MMP) 2 and MMP9 activity and promoted growth, clonogenic survival, migration and invasion of hepatocellular carcinoma (HCC) cells by modulating a tumor suppressor, the tissue inhibitor of metalloprotease 3 (TIM3). Depletion of miR-181b inhibited tumor growth of HCC cells in nude mice [30]. Further studies reported that overexpression of miR-181b could regulate tamoxifen resistance in breast cancer by downregulating TIM3 and facilitating growth factor signaling [31]. Downregulation of miR-181b in human gastric tissues could elevate the expression of cAMP responsive element binding protein1 (CREB1) that suppressed the proliferation and colony formation rate of gastric cancer cells [32]. Together, these findings suggest that miR-181a/b plays an important role in human tumorigenesis.

MiR-181 preferably expresses in hematopoietic cell lineages and is involved in erythropoiesis, granulocytic and megakaryocytic differentiation [33]–[36]. Cuesta et al. [37], found that miR-181a inhibited the translation of the cell cycle inhibitor p27 via 2 functional miR-181a-binding sites in the 3′UTR of p27 and downregulation of miR-181a would cause cell cycle arrest and full differentiation of myeloid cells. MiR-181a could prompt CD4 and CD8 double-positive (DP) T cell development, when ectopically expressed in thymic progenitor [28]. In situ hybridization (ISH) in tonsil tissue sections showed gradual decrease of miR-181b staining intensity from the dark to the light zone in germinal center B cells [38]. These findings indicated the significance of miR-181 in human hematopoietic development.

The importance of miR-181 in hematopoiesis leaded most studies to focus on the role of miR-181 family in hematological malignancies. The pooled HR (0.717, 95% CI:0.631–0.816) of our meta-analysis showed that low level of miR-181a/b expression was significantly relative to poor prognosis in patients with hematological malignancies, suggesting that miR-181a/b might act as tumor suppressor. For example, miR-181a was downregulated in chronic myeloid leukemia (CML) and overexpression of miR-181a effectively suppressed cell growth and induces apoptosis in CML cell line K562 [42]. Downregulation of miR-181a/b resulted in the increasing of TCL1 and BCL1 which are both the lymphoid proto-oncogenes [39], [40]. In line with this, the downregulation of miR-181a in CLL samples also resulted in the significant overexpression of pleomorphic adenoma gene 1 (PLAG1) [41]. The Luciferase reporter and western blot assays had confirmed that RalA was a direct target of miR-181a. However, other studies supported the oncogene role for miR-181a/b. For example, high expression of miR-181a could lead to decreasing of a proapoptotic protein, Bim, in T-cell lymphoma and non-Hodgkin lymphoma cell lines [43], [44]. MiR-181b was downregulated in acute promyelocytic leukemia (APL) cell line NB4 after giving treatment with pharmacological does of all-trans retinoic acid (ATRA) [45], whereas high expression miR-181a could sensitize APL cell lines HL-60 to Ara-C treatment [46]. These paradoxical phenomena could be explained by the fact that ATRA induced APL cells differentiation but Ara-C promoted cells apoptosis. It is still unclear that how miR-181a/b exactly works in hematological cancers. Nevertheless, miR-181a/b could be a useful biomarker at least.

Since miRNAs have unique expression profiles in cancerous samples compared to normal tissue, they are considered as potential biomarkers for prognosis of cancers. We show in here that miR-181a/b is very promising for prognosis prediction in hematological malignancies. Samples of patients in the hematological cancers can be easily gained from peripheral blood, making the feasible life-long monitor of miR-181a/b for those patients. However, several problems should be resolved before miR-181a/b could become a routine clinical application in the future. First, lack of abundant miR-181a/b expression data in global population makes it difficult to set a standard value for the measurement of miR-181/b. Second, a group of miRNAs might be better than a single miRNA. Marccuci et al. [47], detected a set of miRNAs in AML patients (included miR-181a/b) and calculated the miRNAs summary value as a compound predictor to evaluate miRNA expression and the 5 years event-free survivals of patients. More studies should be carried out to compare the prognosis power between miR-181a/b and a group of selective miRNAs.

### Conclusion

Our meta-analysis, representing a quantified synthesis of all published studies of miR-181a/b, has shown that the low-expressed miR-181a/b is significantly associated with poor survival in patients with hematological malignancies. More clinical investigations should be conducted before miR-181a/b can be implemented into the routine clinical management. However, it is still unclear that miR-181a/b acts as a tumor suppressor or as an oncogene. Our study could aid in the delineation of this issue by demonstrating miR-181a/b performance in clinic and provide clues for future investigations.

## Supporting Information

Figure S1 **Target genes of miR-181a and miR-181b.**
(TIF)

Table S1 **Summary table of eligible studies.**
(PDF)

Checklist S1 **PRISMA 2009 checklist.**
(DOCX)
