# Supplementary figures and images for: Prognostic Role of MicroRNA-181a/b in Hematological Malignancies: A Meta-Analysis
Source: PLoS One. 2013 Mar 22;8(3):e59532. doi: 10.1371/journal.pone.0059532 (PMC3606212; doi:10.1371/journal.pone.0059532)

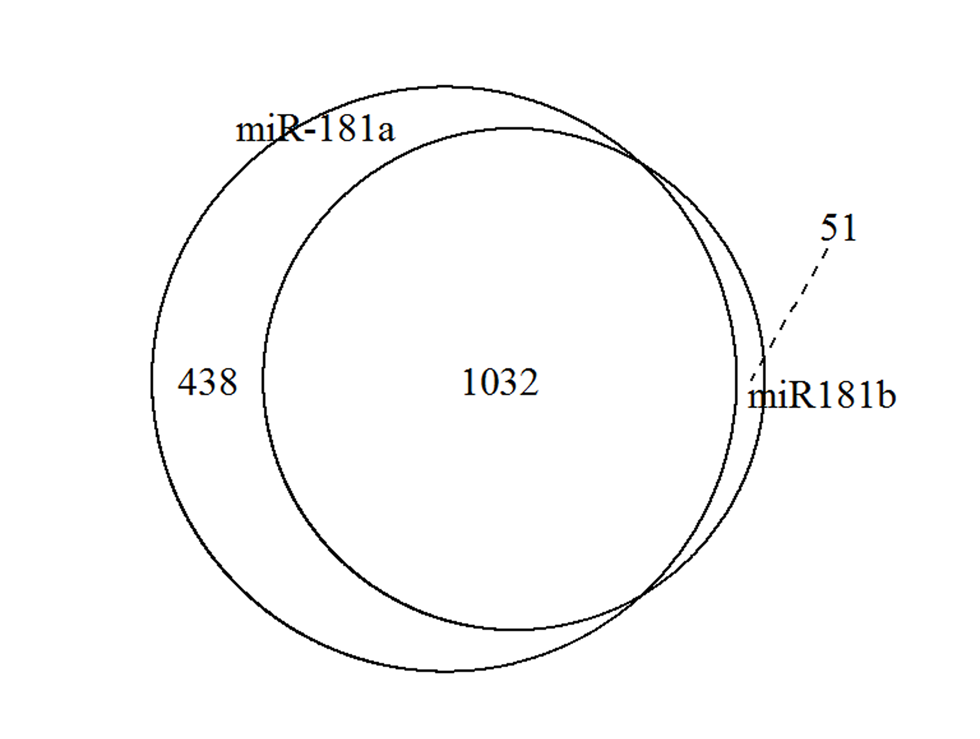

Supplement: Figure S1 — Target genes of miR-181a and miR-181b. (TIF) [file pone.0059532.s001.tif]
