# Supplementary material for: Prognostic Role of MicroRNA-181a/b in Hematological Malignancies: A Meta-Analysis
Source: PLoS One. 2013 Mar 22;8(3):e59532. doi: 10.1371/journal.pone.0059532 (PMC3606212; doi:10.1371/journal.pone.0059532)
Supplement: Table S1 — Summary table of eligible studies. (PDF) [file pone.0059532.s002.pdf]

Table S1. Summary table of eligible studies

Study_id	Publication year	Origin	Study design[a]	Disease[b]	Sample size	miR-181[c]	Dichotomization	Methods[d]	Cut-off	Survival analysis[e]	HR estimation	Follow_up
Schetter.et al.	2008	US	R	colon adenocarcinoma	84	b	high:low	microarray	highest tertile	OS	Reported	68.0(26.0-141.9) months
Gao.et al.	2010	CH	R	NSCLC	47	a	high:Low	TaqMan	median	OS	Reported	high:0-55;low:0-58 months
Rossi.et al.	2010	US	R	CLL	86	b	high:low	TaqMan	median	OS	Reported	20(0-88) months
Schwind.et al.(1)	2010	US	R	CN-AML with FLT3-ITD and/or NPM1wt	122	a	high:low	microarray	median	OS	Reported	6.5(3.10-11.0) years
Schwind.et al.(2)	2010	US	R	CN-AML with FLT3-ITD and/or NPM1wt	122	a	high:low	microarray	median	OS	Reported	estimated 24 months
Zhi.et al.	2010	CH	R	astrocytoma	124	b	high:low	SYBR Green	median	OS	Reported	Training set 34.3(1-98);Validation set 37.1(1-92) months
Jiang.et al.	2011	CH	R	gastric cancer	55	b	high:low	TaqMan	median	OS	Reported	high:1-12.7;low:1-16.6 months
Li.et al.(1)	2012	US	R	CA-AML	33	a/b	high:low	microarray	median	OS	Reported	low:0-5;high 0-5 years
Li.et al.(2)	2012	US	R	CA-AML	53	a/b	high:low	microarray	median	OS	Reported	low:0-4.8;high 0-5 years
Ota.et al.	2011	JP	R	breast cancer	291	a	high:low	TaqMan	highest value of 95% confidence interval	OS	Available data	61(2-90) months
Zhu.et al.	2012	CH	R	CLL	150	a/b	high:low	SYBR Green	mean	OS	Author provided	29(2-160) months
Nakajima.et al.	2006	JP	R	colon cancer	46	b	high:low	TaqMan	3-fold	OS	Survival Curve	high:0-1032;low:0-1400 days
Yang.et al.	2011	TW	R	OSCC	39	a&b	high:low	TaqMan	mean	OS	Reported	22.5±12.5 months

a. Study design: retrospective(R);

b. Disease: NSCLC (non small cell lung cancer), CN-AML (cytogenetical normal acute myeloid leukemia), CA-AML (cytogenetically abnormal myeloid leukemia), CLL (chronic lymphoid leukemia) and OSCC (oral squamous cell carcinoma);

c. MiR-181: a/b represents that miR181-a and miR-181b were tested respectively in the studies, a&b represents miR181a and miR-181b were tested together as a combination;

d. Methods: Both TaqMan and SYBR Green are quantification real time PCR methods;

e. Survival analysis: OS (overall survival).
